# User-initialized active contour segmentation and golden-angle real-time cardiovascular magnetic resonance enable accurate assessment of LV function in patients with sinus rhythm and arrhythmias

**DOI:** 10.1186/s12968-015-0146-9

**Published:** 2015-05-21

**Authors:** Francisco Contijoch, Walter R. T. Witschey, Kelly Rogers, Hannah Rears, Michael Hansen, Paul Yushkevich, Joseph Gorman, Robert C. Gorman, Yuchi Han

**Affiliations:** Department of Bioengineering, University of Pennsylvania, Smilow Center for Translational Research, 3400 Civic Center Blvd, Bldg 421, 7th Floor, Rm 103, Philadelphia, PA 1903 USA; Department of Radiology, University of Pennsylvania, Philadelphia, PA USA; Cardiovascular Division, Department of Medicine, University of Pennsylvania, Philadelphia, Pennsylvania USA; NHLBI, NIH, Bethesda, MD USA; Department of Surgery, University of Pennsylvania, Philadelphia, PA 1903 USA

**Keywords:** Real-time CMR, Arrhythmia, Temporal resolution

## Abstract

**Background:**

Data obtained during arrhythmia is retained in real-time cardiovascular magnetic resonance (rt-CMR), but there is limited and inconsistent evidence to show that rt-CMR can accurately assess beat-to-beat variation in left ventricular (LV) function or during an arrhythmia.

**Methods:**

Multi-slice, short axis cine and real-time golden-angle radial CMR data was collected in 22 clinical patients (18 in sinus rhythm and 4 patients with arrhythmia). A user-initialized active contour segmentation (ACS) software was validated via comparison to manual segmentation on clinically accepted software. For each image in the 2D acquisitions, slice volume was calculated and global LV volumes were estimated via summation across the LV using multiple slices. Real-time imaging data was reconstructed using different image exposure times and frame rates to evaluate the effect of temporal resolution on measured function in each slice via ACS. Finally, global volumetric function of ectopic and non-ectopic beats was measured using ACS in patients with arrhythmias.

**Results:**

ACS provides global LV volume measurements that are not significantly different from manual quantification of retrospectively gated cine images in sinus rhythm patients. With an exposure time of 95.2 ms and a frame rate of > 89 frames per second, golden-angle real-time imaging accurately captures hemodynamic function over a range of patient heart rates. In four patients with frequent ectopic contractions, initial quantification of the impact of ectopic beats on hemodynamic function was demonstrated.

**Conclusion:**

User-initialized active contours and golden-angle real-time radial CMR can be used to determine time-varying LV function in patients. These methods will be very useful for the assessment of LV function in patients with frequent arrhythmias.

## Background

Cine cardiovascular magnetic resonance (CMR) is the gold standard for quantification of ventricular function, however image quality is often compromised in patients with severe rhythm disturbances. To preserve image quality, most 2D multi-slice CMR methods discard data obtained during an arrhythmia, but beat-to-beat variation in left ventricular (LV) function in arrhythmic patients may be important to provide a more accurate estimate of cardiac function and ventricular performance.

Real-time or ‘single-shot’ CMR (rt-CMR) methods have been developed to acquire 2D images continuously, capturing uninterrupted cardiac motion for each heartbeat. Assessment of LV function in normal subjects and patients has been performed using several rt-CMR methods with varying degrees of accuracy, summarized in Table 1 [1–6]. These studies imaged patients in sinus rhythm and a single cardiac cycle from each 2D acquisition was selected for LV function assessment. To date, the potential to measure beat-to-beat variation in LV function has not been described using rt-CMR methods.

**Table 1:** Real-time CMR acquisitions, which measured hemodynamic values and compared results with standard retrospective cine imaging

Publication:	Exposure time (ms)	Frame rate^-1^ (ms)	Trajectory	Parallel imaging	Compressed sensing	Result:
Bauer, et al. I J Cardiovasc Imag 2013 [1]	315	63	Radial	SENSE	No	28 % underestimate
	125	25	Radial			4 % underestimate
Voit, et al. JCMR 2013 [2]	40	40	Radial	SENSE	Yes	10 % underestimate
Muthurangu, et al. Radiology 2008 [3]	40	16	Radial	Yes	No	EF: 1.2 % lower
Boll et al. JCMR 2005 [4]	83	42	Radial	No	No	4 % underestimate
Kuehl, et al. Radiology 2004 [5]	208	100	Radial	No	No	Wall Motion: Comparable
Spuentrup, et al. Radiology 2003 [6]	200	67	Radial	No	No	No Sig Diff
			Spiral			
						No Sig Diff
**Range:**	**40-315**	**16-100**				

The first challenge in systematic measurement of beat-to-beat LV function is that there have been no methods presented for continuous quantitative measurement of LV slice volume during several seconds of MR imaging. A single scan can result in thousands of image frames rendering manual segmentation impractical. As a result, we first sought to develop a quantification method using user-initialized active contours segmentation (ACS) to extract LV slice volume for each image frame, and measure beat-to-beat slice LV function in human subjects [7, 8]. We validated our approach via comparison to manual segmentation on clinically accepted software.

The second challenge is the determination of adequate temporal resolution for capturing beat-to-beat differences. Temporal resolution, in non-view sharing techniques, describes both the image exposure time (T_ex_) and the reconstructed image frame rate (FR). However, when view sharing is performed, these two parameters can be evaluated separately. Recent reconstruction techniques have increased achievable undersampling and have led to improved temporal resolution (Table 2) [7, 9–23]. However, there has been a lack of consensus regarding the image exposure time and image frame rates necessary for accurate measurement of cardiac motion and function

**Table 2:** Recent improvements in image reconstruction have led to an array of real-time CMR publications

Publication:	Exposure time (ms)	Frame rate^-1^ (ms)	Trajectory	Parallel imaging:	Compressed Sensing:
Li, et al. MRM 2013 [9]	30	30	Radial	SENSE	Yes
Lurz, et al. Eur Heart Journal 2012 [10]	35	35	Radial	SENSE	No
Jones, et al. JMRI 2011 [11]	35	35	Radial	SENSE	No
Seiberlich, et al. MRM 2011 [12]	35	35	Spiral	GRAPPA	Yes
	18	18			
Seiberlich, et al. MRM 2011 [13]	35	35	Radial	GRAPPA	No
Saybisili, et al. MRM 2010 [14]	151	151	Radial	GRAPPA	No
Uecker, et al. NMR Biomed 2010 [15]	20	30	Radial	SENSE	Yes
Uecker, et al. MRM 2010 [16]	250	50	Radial	SENSE	No
	90	18			
Zhang, et al. JCMR 2010 [17]	52	52	Radial	SENSE	Yes
	22	22			
Zhang, et al. JMRI 2010 [18]	250	50	Radial	No	No
Lurz, et al. JMRI 2009 [19]	35	35	Radial	Yes	No
Sorensen, et al. IEEE TMI 2009 [7]	125	125	Radial	Yes	No
	50	50			
Winkelmann, et al. IEEE TMI 2007 [20]	603	2.59	Golden	No	No
	88	2.6			
Spuentrup, et al. Invest Radiol 2003 [21]	200	100	Radial	No	No
			Spiral		
Larson, et al. MRM 2001 [22]	91	45	Radial Multi-Echo	No	No
Shankaranarayanan, et al. Radiology 2001 [23]	150	55	Radial	No	No

We utilized the flexibility of golden-angle imaging to explore the impact of these two parameters (T_ex_ and FR) on measured LV slice function. We reconstructed 2D rt-CMR data with varying number of projections to determine the trade-off between image quality and endocardial border blurring. Varying image frame rates allowed us to explore the trade-off between temporal spacing of LV slice volume measurements and the dataset size. The impact of these two basic image parameters was evaluated in clinical patients using measured LV slice volume. Finally, a preliminary study of patients with arrhythmias illustrates the varying hemodynamic function that can be observed with this technique.

## Methods

### Patients

The study was approved by our Institutional Review Board and all subjects (N = 22, 44.7 ± 16.0 years old and 55 % male) gave written informed consent. 18 subjects were in sinus rhythm and did not have rhythm disturbances during scanning. 4 patients had frequent rhythm disturbances where premature ventricular contractions occurred at least as frequently as every 4^th^ beat. All patients were referred for assessment of non-ischemic cardiomyopathy: premature ventricular contractions (PVC) (n = 8), rule out sarcoid (n = 3), hypethrophic cardiomyopathy (HCM) (n = 2), arrhythmogenic right ventricular dysplasia (ARVD) (n = 2), nonspecific cardiomyopathy (n = 6), pulmonary hypertension (n = 1).

### Image acquisition

CMR was performed on a 1.5 T clinical imaging system (Avanto, Siemens Healthcare, Erlangen, Germany) equipped with nominal 40 mT/m magnetic field gradients, body RF transmit and a 32-channel, anterior and posterior RF receiver array.

Cine-CMR was obtained using a conventional 2D, breath-held, multi-slice, retrospectively-gated, balanced steady-state free-precession (bSSFP) sequence with the following imaging parameters, TE = 1.12-1.31 ms, TR = 2.24-2.62 ms, matrix = 144-192 x 192, FOV = 240-320 mm x 240-400 mm, pixel size = 1.25 – 2.08 x 1.25 - 2.08 mm, BW = 930 Hz/pixel, phases = 30, slices = 12-16, slice thickness = 8 mm, slice spacing = 10 mm and temporal resolution = 29.8 ± 7.0 ms.

Real-time data was obtained using a 2D, multi-slice, free-breathing bSSFP sequence with a golden-angle radial trajectory with the following imaging parameters, TE = 1.4 ms, TR = 2.8 ms, number of radial k-space data = 128, FOV = 220 mm - 300 mm, pixel size = 1.72 – 2.34 x 1.72 – 2.34 mm, bandwidth = 1184 Hz/pixel, slice thickness = 8 mm, and k-space sampling according to the golden-angle Φ = 111.25*°*. Imaging was performed in the short axis of the left ventricle over the same volume as cine-CMR by copying slice positions acquired in cine-CMR. 4000 radial projections (11 s) per slice were acquired in sinus rhythm. 6000 radial projections (17 s) per slice were acquired in patients with arrhythmia.

### Reconstruction of real-time CMR

**Fig. 1:**
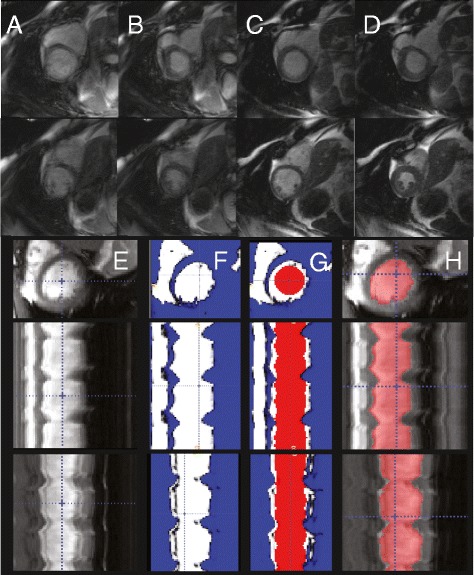
Example of image quality and visual depiction of quantification process. Two slice locations imaged during severe arrhythmia demonstrate the corruption of conventional CMR during **A** end-diastole and **B** end-systole. Real-time imaging does not demonstrate corruption at **C** end-diastole and **D**) end-systole. Panels **E**-**H** illustrate the active contour segmentation (ACS) process. The short axis image is shown in the top row. The second and third rows illustrate projections along time. **E** Image sequences are loaded as 3D volumes (2D + t) into software. **F** The user defines an intensity threshold, which defines the endocardial border and results in a feature image. **G** Region growing is then performed inside the feature image. **H** The resulting segmentation can be manually corrected for any errors

Real-time image reconstruction was performed offline using a non-Cartesian SENSE algorithm in open-source software [7, 24, 25]. Examples of the image quality of conventional cine (**A-B**) and the real-time reconstruction with 34 radial projections (**C-D**) in a patient with frequent rhythm disturbances are shown in Fig. 1. We reconstructed data with maximum view sharing, resulting in one image frame for each radial projection. For the highest image frame rate, there were approximately 4,000 images for each 11-s acquisition per slice location. Reconstruction of a complete multi-slice dataset took approximately 45 min for 34 radial projections per image with a 357 frames per second (fps) reconstructed frame rate on a workstation with a single graphics processing unit (GPU).

### Conventional hemodynamic quantification

Gold-standard volumetric measurements were obtained using JCMR/SCMR 2013 guidelines for manual segmentation in clinically accepted software (Qmass, Medis, Netherlands). Detailed tracing of the endocardial border in standard 2D multi-slice, cine-CMR images yielded end-diastolic and end-systolic LV slice volume (EDV and ESV) estimates for each slice (slice area multipled by slice thickness). Papillary muscles were excluded from segmentations and resulting volume estimates. Volumetric quantification of two cardiac phases was obtained in approximately 10-15 min per patient. Global measurements of EDV and ESV were obtained by summation of of slice volumes from the short-axis stack.

### Active contour segmentation (ACS)

Quantification of slice volume in all image frames of a 2D acquisition was performed offline through user-initialized active contour segmentation (ACS) (ITK-SNAP, University of Pennsylvania, Philadelphia, PA) as detailed in Fig. 1**(E-H)** [8]. 2D image data was arranged in a stack Nx x Ny x Nt; the typical size was 128 x 128 x 4,000. Manual intensity thresholding was used to generate a set of feature images of the LV intraventricular volume. Ventricular segmentation was automatically initialized using a 3 x 3 x Nt pixel column centered in the ventricle. Active contour segmentation was performed using region competition with user-defined, patient- and slice-specific balloon and curvature forces [26]. The advantage of this arrangement was that contours between consecutive image frames were smooth, resulting in temporally consistent and smooth LV slice area. Papillary muscles were excluded from the segmentation and volume calculation using the feature image and, if necessary, manual correction. Typically, 1-2 slices per patient would require manual correction due to LV outflow tract, papillary muscle, or radial streak artifact. Using ACS, segmentation takes approximately 3 min for a single slice and 30 min for the entire LV (all slices and all phases). LV slice volume was quantified by multiplying the slice area multiplied by slice thickness and slice ED and ES volumes were automatically determined from local maxima and minima using a peak detection algorithm (Matlab, The MathWorks, Natick, MA). Global EDV, ESV, stroke volume (SV), and ejection fraction (EF) were measured by summation of measured slice EDV and ESV values.

### Effect of image parameters on measured LV function

To determine the effect of imaging parameters on measured LV slice function, we reconstructed the same golden-angle radial data at different temporal resolutions. Although there are a large number of potential reconstruction parameters that contribute to temporal resolution, we focused on the image exposure time *T*_*ex*_ and frame rate *FR*.

In this image acquisition, image exposure time is the number of radial projections used to reconstruct a single image frame and is analogous to camera exposure time. Like camera exposure time, increasing the number of radial projections results in high image signal-to-noise ratio (SNR). However, it also introduces blurring due to cardiac motion. Increasing the exposure time leads to blurry endocardial wall boundaries, which compromises endocardium visualization.

The image frame rate was defined as the number of image frames per second. It was possible for the frame rate to exceed the exposure time because a single projection could be shared in more than one image frame. The maximum possible frame rate, with view sharing is determined by the TR of the bSSFP sequence (TR = 2.8 ms leads to a FR = 357 fps). Decreasing the amount of view sharing decreases the size (and time) of the reconstruction and reduces the number of images for segmentation. However, it could result in undersampling of the slice volume curves leading to errors in both volume and time detected for slice EDV and ESV values.

Seven datasets in normal sinus rhythm were used to investigate the sensitivity of measured LV function to changes in exposure time and frame rate. These patients had exact agreement in slice location between cine and real-time images. The patients had a range of normal heart rates (54 - 86 bpm). For each patient, a single mid-ventricular slice location acquired using both real-time and cine imaging was selected for analysis. Exposure time was varied by reconstructing images from *N*_*p*_ = 10-300 radial projections, corresponding to an exposure time *T*_*ex*_ = TR*N_*p*_ = 28-840 ms. The image frame rate was evaluated by modifying the number of shared projections at fixed exposure time *T*_*ex*_ = 95.2 ms. This resulted in image data with display frame rates FR of 1.2 - 357 fps. For each dataset, ACS was performed to measure time-varying slice volume and the relationship between measured slice EDV, ESV, SV, and EF was quantitatively compared to manual segmentation of the corresponding cine-CMR slice.

### Statistical analysis

A two-tailed paired Student’s *t*-test (p <0.05) was used to detect significant differences in hemodynamic values between the three different approaches (cine with QMass, cine with ACS, and rt-CMR with ACS). A Bland-Altman test was performed to measure differences in measured hemodynamics between the approaches. A two-tailed paired Student’s *t*-test (p < 0.05) was used to detect significant differences in slice hemodynamic values at different image exposure times and frame rates.

## Results

### Validation of active contour segmentation (ACS)

**Fig. 2:**
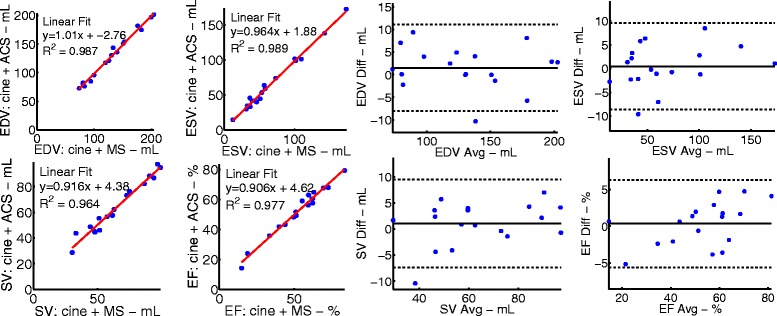
Consistency between global LV function measured using manual and semi-automated segmentation (ACS) of standard retrospectively gated cine images. **Left** Correlation between end-diastolic volume (top left), end-systolic volume (top right), stroke volume (bottom left), and ejection fraction (bottom right) obtained via manual segmentation (MS) using QMass and ACS. **Right** Bland Altman analysis for the same measurements

To validate the use of ACS, we processed cine-CMR images with ACS and compared measured LV values to those obtained by manual segmentation of the same cine-CMR images. The results from 18 clinical patients in sinus rhythm are shown in Fig. 2. The patients had a range of global volume and function (EDV range = 80 - 204 mL, ESV range = 31 - 174 mL, EF range = 14.8 - 72.7 %). The same cine-CMR images were processed using the two segmentation methods and there was no significant difference in measured global EDV (p = 0.20), ESV (p = 0.66), SV (p = 0.32), or EF (p = 0.63). Bland-Altman analysis shows negligible biases, which are not statistically significant (bias: EDV = 1.5 mL, ESV = 0.5 mL, SV = 1.0 mL, and EF = 0.3 %).

### Influence of image exposure time and frame rate on measured LV function

**Fig. 3:**
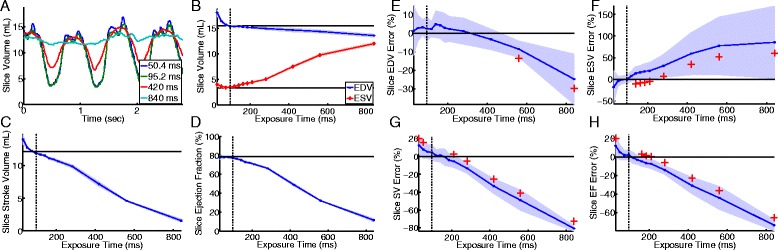
Effect of exposure time on measured LV function. Panels **A**-**D** illustrate the effect of exposure time on measured slice volume in one patient. **A** Measured LV slice volumes for 4 exposure times (50-840 ms) in a clinical patient in sinus rhythm with a heart rate of 76 bpm. **B** Dependence of measured end-diastolic (blue) and end-systolic (red) slice volumes on exposure time. **C** The effect of exposure time on measured stroke volume in a slice. **D** The effect of exposure time on measured ejection fraction in a slice. The corresponding cine slice measurements are depicted with a horizontal black line in **B**, **C** and **D**. The real-time values depict the mean (solid line) and standard deviation (shaded area) of the volume measured across real-time beats. Panels **E**-**H** illustrate the error between manual segmentation of cine images and semi-automated processing of real-time images from single slices in 7 different patients. The change in the measured error (blue line) and standard deviation (blue shaded area) as a function of exposure time are shown for measured slice EDV (**E**), ESV (**F**), SV (**G**), and EF (**H**). An exposure time of 95.2 ms resulted in no statistical difference of the measured error (vertical black dotted line). The occurrence of a statistically significant difference is shown in each panel with red crosses

After validation of ACS, we determined the effect of image exposure time on measured LV slice function by reconstructing golden-angle radial real-time data using a varying number of radial projections. We performed ACS on a single slice in seven clinical patients in sinus rhythm with heart rates in normal physiologic range (54 - 86 bpm). Comparison of measured slice EDV, ESV, SV, and EF to the corresponding cine slice values allowed for the percent error of real-time measurements with respect to the manually segmented cine data to be calculated and averaged among the patients. Fig. 3 shows results from one subject (**A-D**) as well as the error observed from the seven subjects (**E-H**). In all patients, there was a progressive reduction in measured slice EDV and SV and an increase in slice ESV with increasing exposure time, consistent with a blurring of cardiac motion, specifically blurring of the endocardial wall. We found that an exposure time of 95.2 ms (34 radial projections) resulted in the best LV volume accuracy as compared to cine. Higher exposure times resulted in loss of accuracy due to cardiac blurring while lower exposure time results in loss of accuracy due to increased undersampling artifacts and loss of signal-to-noise ratio which makes accurate segmentation challenging (seen in the increased error bars).

**Fig. 4:**
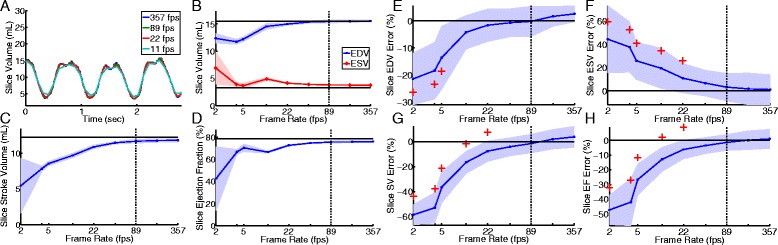
Effect of image frame rate on measured LV function. Panels **A**-**D** illustrate the effect of frame rate on measured slice volume in one patient. **A** Measured LV volumes for 4 frame rates 11, 22, 89, and 357 fps in a single clinical subject in normal sinus rhythm (heart rate = 76 bpm) from images with an exposure time of 95.2 ms. **B** Dependence of measured end-diastolic (blue) and end-systolic (red) slice volumes on frame rate. **C** Effect of image frame rate on measured stroke volume in a slice. **D** Effect of frame rate on measured ejection fraction in a slice. The corresponding cine slice measurements are depicted with a solid black line in **B** **C**, and **D**. Real-time measurements are shown with the mean (solid colored line) and standard deviation (shaded area) obtained from several beats during the acquisition. Panels **E**-**H** illustrate the mean error between manual segmentation of cine images and semi-automated processing of real-time images from single slices in 7 different patients. The mean error (blue line) and standard deviation (shaded area) as a function of frame rate is shown for measured slice EDV (**E**), ESV (**F**), SV (**G**), and EF (**H**). The hemodynamic values show statistically significant errors (red cross) at frame rates lower than 44 fps. The black dotted line at 89 fps illustrates the close agreement in hemodynamic values

We next determined the effect of image frame rate on measured LV slice volume by varying the number of shared radial projections. Results from one subject (**A-D**) and group values (**E-H**) appear in Fig. 4. As the frame rate decreases, the size of the dataset processed decreased and the volume curve was increasingly undersampled, which resulted in errors in both time and volume when selecting maximum and minimum volumes. Our findings indicate that frame rates above 89 fps provide accurate measurement of LV slice volumes relative to values obtained via manual segmentation of cine-CMR for all observed heart rates.

### Validation of the adequacy of real-time imaging for volume assessment

**Fig. 5:**
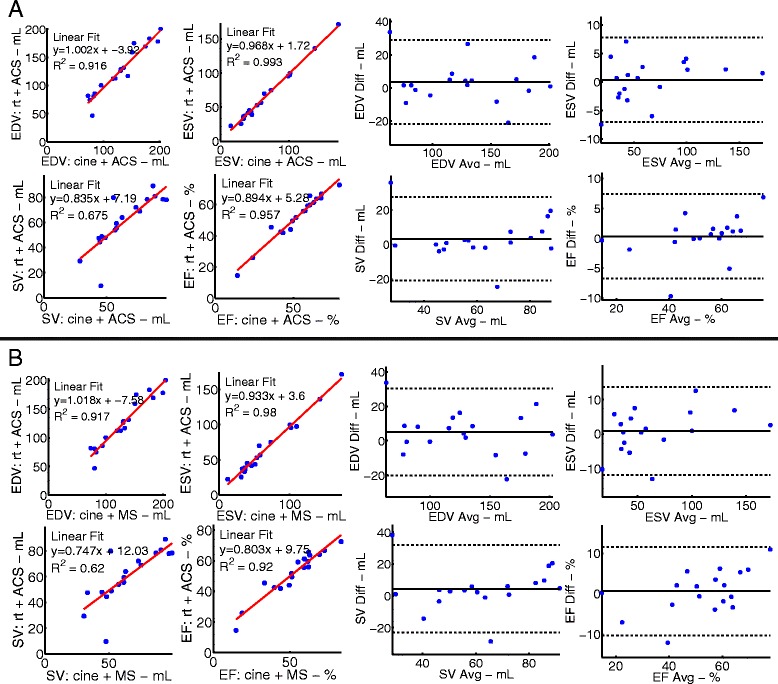
Consistency between global LV function measured using manual and semi-automated segmentation (ACS) of both cine and real-time datasets. Panel **A** illustrates the consistency in measured hemodynamic values obtained from ACS in cine and real-time images. **Left** Correlation between global end-diastolic volume (top left), end-systolic volume (top right), stroke volume (bottom left), and ejection fraction (bottom right) obtained via ACS of cine and real-time image sets. **Right** Bland Altman analysis of the measurements. Panel **B** illustrates the agreement between LV function measured via manual segmentation of cine images and ACS of real-time image data **Left** Correlation between end-diastolic volume (top left), end-systolic volume (top right), stroke volume (bottom left), and ejection fraction (bottom right). **Right** Bland Altman analysis of the measurements

Despite variation in FOV, the difference in pixel length or pixel area between cine and real-time acquisitions was not statistically significantly different (paired *t*-test yielded p-values of p = 0.25 and p = 0.97, respectively). Using the exposure time and frame rate described above, global LV volume measurements made using real-time images and ACS were compared to those obtained from cine images using ACS (Fig. 5A). There was no significant difference in global EDV (p = 0.24), ESV (p = 0.69), SV (p = 0.26), or EF (p = 0.72). Bland-Altman analysis shows negligible biases, which are not statistically significant (EDV = 3.7 mL, ESV = 0.4 ml, SV = 3.3 mL, and EF = 0.3 %).

Furthermore, measurements using the real-time image acquisition and ACS were compared to manual segmentation of cine-CMR images (Fig. 5B). Again, there was no significant difference in global EDV (p = 0.10), ESV (p = 0.58), SV (p = 0.20), or EF (p = 0.62). Bland-Altman analysis shows negligible biases, which are not statistically significant (bias: EDV = 5.2 mL, ESV = 0.8 mL, SV = 4.3 mL, and EF = 0.6 %).

### Evaluation of time-varying LV slice volumes

**Fig. 6:**
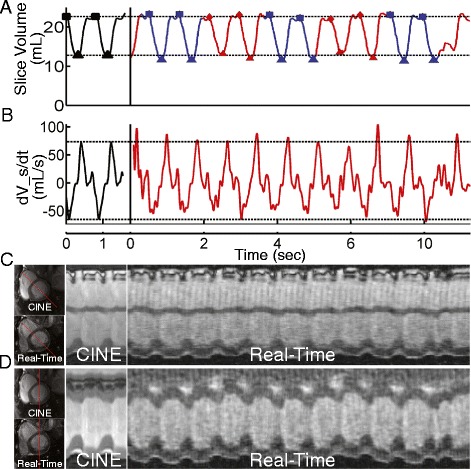
Cine and real-time LV slice volumes and volume rates in a single clinical patient in predominantly sinus rhythm (heart rate = 76 bpm). **A** Single-slice volume over time for cine (black) and real-time (red = end-expiration, blue = inspiration). In the cine images, two cardiac cycles are shown by repeating the image data to observe the continuity in volume around end-diastole. Slice end-diastolic volume (EDV) (square) and end-systolic volume (ESV) (triangle) points were identified in both cine and real-time data for volumetric comparison. Slice EDV and ESV from manual segmentation of cine images are plotted as dashed horizontal lines. Respiratory phase was determined by tracking diaphragm motion. **B** Slice Volume change rate (dV_s/dt) for cine and real-time data. Maximum and minimum dV_s/dt from cine frames are plotted as dashed horizontal lines. **C** 1D projections through cine and real-time images, with the line indicating the projection location which intersects the anteroseptum and inferolateral wall, and **D** 1D projections through cine and real-time images, with the line indicating the projection through the anterior and inferior walls. In the real-time data, a single arrhythmic event can be seen (t = 10 sec)

Cine (black) and real-time (blue/red) slice volume measurements at a representative slice position are shown in Fig. 6A. There was close agreement between the slice volume curve shape (normalized correlation coefficient = 0.96) as well as measured slice end-systolic and end-diastolic values. In all patients, the slice volume was affected by respiratory motion. Since cine images were acquired at end-expiration, volumes obtained via real-time during end-expiration were utilized for volumetric comparison (red). Periods of end-expiration were identified based on the diaphragm motion observed in the images. A single PVC was observed at approximately t = 10 sec. The ability of this technique to accurately capture the change in slice volume (dV_s/dt) throughout the cardiac cycle is shown in the Fig. 6B (correlation coefficient = 0.90). The maximum and minimum dV_s/dt observed from cine values agree with those obtained during the real-time scan. Furthermore, the features of the curve were similar in both techniques, with the most rapid change in LV slice volume occurring during ventricular relaxation. The change in volume demonstrates that the real-time method can accurately measure the change in slice filing and ejection. Fig. 6C and D show 1D projections through the left ventricle for both methods along two different directions.

### Acquisition of real-time images in patients with persistent rhythm disturbances

**Fig. 7:**
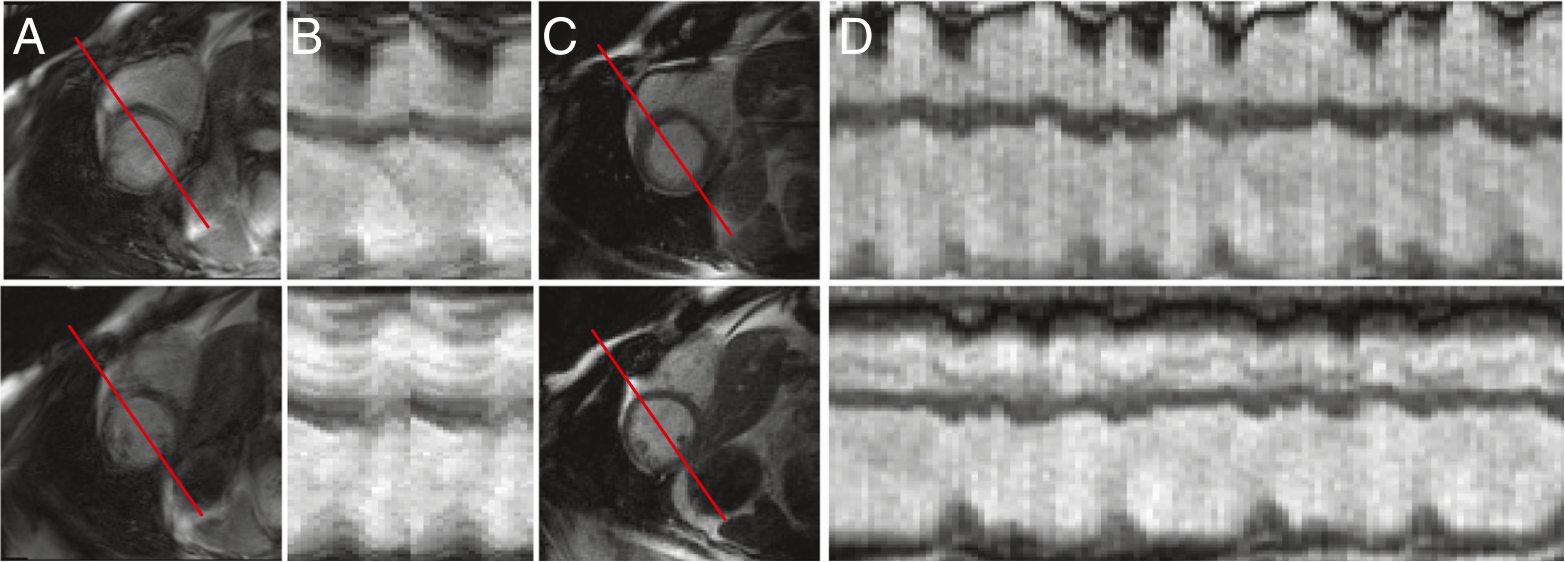
Comparing cine acquisition with real-time acquisition in a patient with severe rhythm disturbances at two slice locations (top and bottom) **A** Cine acquisitions with the line indicating the location of 1D projection. The images are blurred due to acquisition during ectopic beats **B** 1D projection shows temporally blurred cine acquisition due to errors in ECG-gating. **C** Real-time end-diastolic images at two corresponding slice locations. The images are free from corruption due to ectopic beats. **D** The real-time acquisition shows the frequency of ectopic beats with no spatial or temporal blurring

**Fig. 8:**
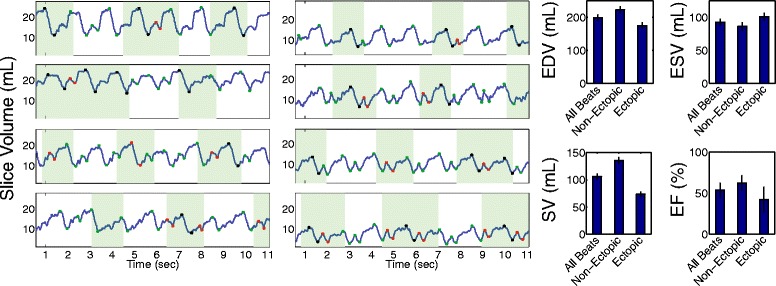
Analysis of one patient with frequent PVCs. Left) Slice volume over time curve of all short axis slices from base to apex. The slice volume curve allows for separation of end-diastolic and end-systolic slice volumes from PVC contractions (red points within the green boxes) from non-ectopic end-diastolic volume (black points within green boxes). Green boxes illustrate end-expiration period of respiration based on visualization of the diaphragm. Right) Volumetric analysis that included all beats (ectopic and non-ectopic), only ectopic beats, and only non-ectopic beats

After validating the ACS quantification and real-time imaging parameters in patients without rhythm disturbances, 4 clinical patients with rhythm disturbances were imaged. For one of these subjects, the cine images obtained at end-diastole as well as a projection through the heart over the cardiac cycle are shown in Fig. 7A-B. Real-time imaging allows for severe multi-focal PVCs to be visualized along the projection (Fig. 7C-D). In addition to obtaining images of high quality for clinical interpretation, quantification of slice volume from real-time images via ACS permitted evaluation of the effects of the rhythm disturbances on ventricular volumes. Slice-by-slice analysis of ventricular volumes of another patient is shown in Fig. 8. For each slice location, the observed slice EDV and ESV varied depending on the timing of the PVC. To obtain global volumetric values, the average slice EDV and SV for each beat morphology (within the end-expiratory period) were summed across slice locations. End-expiratory periods were identified by visual observation of diaphragm motion. As a result, volumes from different slices were combined to characterize the same type of beats. For this patient, global EDV, ESV, SV, and EF from all beats, ectopic beats, and non-ectopic beats are shown in bar graphs. Table 3 shows the values for all four arrhythmic subjects.

**Table 3:** Variation in volumetric evaluation due to ectopic contractions

Subject:	End-diastolic volume (mL)			End-systolic volume (mL)			Ejection fraction (%)		
	All	Non-Ectopic	Ectopic	All	Non-Ectopic	Ectopic	All	Non-Ectopic	Ectopic
1	197.5 ± 8.4	231.4 ± 9.9	165.6 ± 7.3	95.1 ± 4.4	84.4 ± 4.4	106.6 ± 4.5	51.9 ± 4.3	63.5 ± 9.2	35.7 ± 9.6
2	133.9 ± 5.7	149.4 ± 5.9	114.8 ± 5.2	76.3 ± 4.2	69.7 ± 3.8	84.9 ± 5.1	43.0 ± 11.4	53.3 ± 8.4	26.0 ± 14.9
3	113.8 ± 6.9	128.3 ± 7.6	88.7 ± 6.2	56.4 ± 4.6	57.9 ± 4.7	54.9 ± 4.5	50.5 ± 8.8	54.9 ± 8.2	38.2 ± 13.5
4	123.0 ± 6.7	130.3 ± 7.3	104.1 ± 5.8	43.1 ± 4.1	44.9 ± 4.5	42.1 ± 2.4	65.0 ± 12.2	65.5 ± 13.4	59.6 ± 9.6

## Discussion

We proposed and validated an active contour segmentation (ACS) algorithm for measurement of time-varying LV slice volume and estimation of global LV function. We used this method to determine the impact that two key components of temporal resolution, image exposure time and frame rate, have on accurate quantification of LV function by reconstructing golden-angle radial data at multiple image exposure times and frame rates. Furthermore, we illustrate that our method of real-time imaging combined with ACS, can provide estimates of time-varying global LV function during severe arrhythmias.

For nearly all types of CMR sampling trajectories, resolution must be determined *a priori*, so it is not known whether spatial, temporal resolution and signal-to-noise ratio are optimal or if Nyquist undersampling artifacts are minimized. The golden-angle radial trajectory is one exception; it allows for reconstruction of images from any number of radial views and a large number of these radial views are guaranteed not to overlap, permitting uniform k-space sampling density, regardless of the number of projections chosen for reconstruction [20]. Like all radial trajectories, view sharing is also possible. Overall, the flexibility to control both exposure time and frame rate resulted in a large continuum of possible image reconstructions with varying temporal resolution, for which the accuracy of a real-time scan could be determined retrospectively.

As reported in Tables 1 and 2, there are a number of previous rt-CMR studies that employed considerable variability in exposure time and frame rate. Our results indicate that golden-angle radial sampling permits accurate measurement of LV volume with image exposure times of 95 ms at high image frame rates (>89 fps).

Bauer et al showed that imaging with an exposure time of 315 ms and frame rate of 16 fps or exposure time of 125 ms with frame rate of 40 fps does not result in accurate LV estimation and our results agree with this evaluation [1]. Voit et al used a much shorter exposure time (40 ms) and frame rate of 25 fps and found an underestimation of EF [2]. For our reconstruction, the loss of image quality associated with a 40 ms exposure time prohibited robust quantification at all slice positions. It is possible that the underestimation reported was due to a lack of image quality and the use of view sharing to increase the exposure time while maintaining a high frame rate may allow for more accurate volumetric evaluation.

The image exposure time we found is longer than recent publications, but it may be advantageous due to the higher SNR [9–13, 15–19]. Although, the long exposure time may introduce errors during rapid wall motion, the use of a very high image frame rate (>89 fps) may limit these errors. The exposure time and frame rate we determined may be impacted by several elements of our approach including the radial k-space trajectory, the spatial resolution (128 points per readout), and quantification method (semi-automated extraction of slice volume). For different approaches including spiral or Cartesian imaging, compressed-sensing reconstructions, higher spatial resolution, or different imaging tasks such as wall motion abnormality imaging, a more restrictive set of parameters may be necessary.

As discussed in Setser et al, the optimal rate to sample the left ventricular volume curve must be sufficient to capture the highest frequency cardiac motions [27]. They reported 40 ms sampling rate (frame rate^-1^) was sufficient for measurement of LV function at heart rates up to 100 bpm. Our findings (>89 fps) indicate a higher frame rate is necessary. This might be due to the interplay between frame rate and image exposure time or the impact of radial k-space sampling. Although a higher frame rate is desirable for accurate sampling the LV volume curve, it directly impacts the size of the reconstruction problem and necessary computational power. As a result, the determination of the minimum frame rate necessary is important for online reconstructions as well as subsequent post-processing steps.

An additional advantage of retrospective reconstruction of image frames is that the reconstruction can be adapted to changes in heart rate during pharmacologic or exercise stress testing, although the relationship between exposure time, frame rate and LV function should again be determined.

One limitation of this study is that temporal filtering or regularization of either k-space data or image frames were not used, since this would complicate interpretation of the temporal resolution [28]. However, the framework presented offers an approach to measure the impact of these techniques.

One major impediment to routine use of real-time imaging is the task of synchronizing data obtained from different slices to obtain meaningful cardiac volumes. In this method, we use the maximum and minimum slice volume. Previous studies have utilized this assumption [1, 2, 4, 6] and in this work, we are able to demonstrate that the difference from cine-CMR is small, as shown in Fig. 2.

For real-time imaging to replace conventional cine acquisitions, other capabilities beyond volumetric quantification such as the evaluation of regional wall motion abnormalities, are necessary. Unfortunately, we are unable to evaluate this in our patient population due to lack of regional wall motion abnormalities. New constraints and future work is needed to evaluate regional wall motion.

It is possible that the heart position varied between breathheld acquisitions (cine imaging) and end-expiration during free breathing (real-time imaging). This potential source of error is included in the comparison of cine + ACS and rt + ACS and no significant differences in measured volumes were observed.

Conventional cine CMR may be badly corrupted during ectopy, which necessitates the use of arrhythmia rejection techniques that may not work well. As a result, real-time methods are essential in patients with severe arrhythmia to characterize LV function. Real-time imaging also allows for imaging of ectopic beats and we present an initial quantitative beat-to-beat analysis of time-varying LV function in patients with severe arrhythmia where characterization of sinus, PVC, and post-PVC beats become possible. In patients with sinus rhythm, beat-to-beat left and right ventricular volumes obtained during changes of respiration might be important in diseases such as constrictive pericarditis.

## Conclusion

In conclusion, we demonstrated that 2D golden-angle rt-CMR combined with ACS can be used to obtain accurate slice volume analysis in clinical patients in sinus rhythm. The use of LV slice volume (and measures such as slice EDV, ESV, SV, and EF) to evaluate imaging and reconstruction parameters provides a quantitative and physiologically-based approach to evaluate parameters of different imaging techniques. Initial results obtained from patients with severe rhythm disturbances illustrate the clinical potential of this 2D method to obtain time-varying LV function. Future work is planned to improve this technique to combine ECG information with image frames as well as to explore simultaneous acquisition of multiple slices.

## Abbreviations

CMR: Cardiovascular magnetic resonance
rt-CMR: Real-time cardiovascular magnetic resonance
LV: Left ventricular
ACS: Active contour segmentation
*T*_*ex*_: Image exposure time
*FR*: Image frame rate
PVC: Premature ventricular contraction
HCM: Hypertrophic cardiomyopathy
ARDV: Arrhythmogenic right ventricular dysplasia
bSSFP: Balanced steady-state free-precession
TE: Echo time
TR: Repetition time
BW: Bandwidth
FOV: Field of view
Fps: Frames per second
GPU: Graphics processing unit
EDV: End-diastolic volume
ESV: End-systolic volume
SV: Stroke volume
EF: Ejection fraction
SNR: Signal-to-noise ratio
